# Indirect Vision-Based Localization of Cutter Bolts for Shield Machine Cutter Changing Robots

**DOI:** 10.3390/s25247685

**Published:** 2025-12-18

**Authors:** Sijin Liu, Zilu Shi, Yuyang Ma, Yang Meng, Jun Wang, Qianchen Sha, Yingjie Wei, Xingqiao Yu

**Affiliations:** 1 China Railway 14th Bureau Group Corporation Limited, Jinan 250101, China; ahlsj@126.com (S.L.); wangjunztj@163.com (J.W.); 15851885450@163.com (Q.S.); wyj_crcc14_2022@outlook.com (Y.W.); 17786291229@163.com (X.Y.); 2 School of Mechanical Engineering, Dalian University of Technology, Dalian 116024, China; szl_dlut@mail.dlut.edu.cn (Z.S.); mengyang@mail.dlut.edu.cn (Y.M.)

**Keywords:** shield cutters changing robot, visual localization, non-overlapping point cloud registration, image segmentation

## Abstract

In operations involving the replacement of shield machine disc cutters, challenges such as limited space, poor lighting, and slurry contamination frequently lead to occlusions and incomplete data when using direct point cloud-based localization for disc cutter bolts. To overcome these issues, this study introduces an indirect visual localization technique for bolts that utilizes image-point cloud fusion. Initially, an SCMamba-YOLO instance segmentation model is developed to extract feature surface masks from the cutterbox. This model, trained on the self-constructed HCB-Dataset, delivers a mAP50 of 90.7% and a mAP50-95 of 82.2%, which indicates a strong balance between its accuracy and real-time performance. Following this, a non-overlapping point cloud registration framework that integrates image and point cloud data is established. By linking dual-camera coordinate systems and applying filtering through feature surface masks, essential corner coordinates are identified for pose calibration, allowing for the estimation of the three-dimensional coordinates of the bolts. Experimental results demonstrate that the proposed method achieves a localization error of less than 2 mm in both ideal and simulated tunnel environments, significantly enhancing stability in low-overlap and complex settings. This approach offers a viable technical foundation for the precise operation of shield disc cutter changing robots and the intelligent advancement of tunnel boring equipment.

## 1. Introduction

As urban underground space development continues to expand, shield machines have emerged as essential equipment for the construction of subways, highways, water conveyance tunnels, and utility corridors. Disc cutters, a critical component of the shield machine, experience significant wear due to prolonged exposure to high stress, fluctuating loads, and corrosive environments [1], which necessitates frequent inspection and replacement. In some complex tunneling projects, the time allocated for disc cutter inspection and replacement can represent nearly one-third of the overall construction timeline. Currently, these operations predominantly rely on manual labor conducted at the rear of the disc cutter head, located in a high-temperature, high-pressure, and high-humidity environment at the tunnel face. Each disc cutter typically weighs over 200 kg and features a complex fastening mechanism, creating substantial safety hazards during manual replacement.

It is essential to advance the intelligent enhancement of tunnel boring machinery and to develop automated disc cutter replacement technologies utilizing robotic systems. Currently, numerous construction companies and research institutions within the tunneling sector are actively pursuing the research and development of shield disc cutter changing robots to address the increasing demand for automated construction technologies [2,3]. However, a significant challenge in replacing manual labor with automated systems is the robot’s ability to perceive its environment. In comparison to sensing methods like LiDAR, vision-based perception systems present several advantages: they are more cost-effective, capable of conducting high-density measurements in targeted areas, provide richer information, and exhibit greater adaptability to various operational requirements. As a result, machine vision has emerged as a crucial enabling technology for the development of environmental perception systems in automated disc cutter replacement equipment [4].

**Figure 1:**
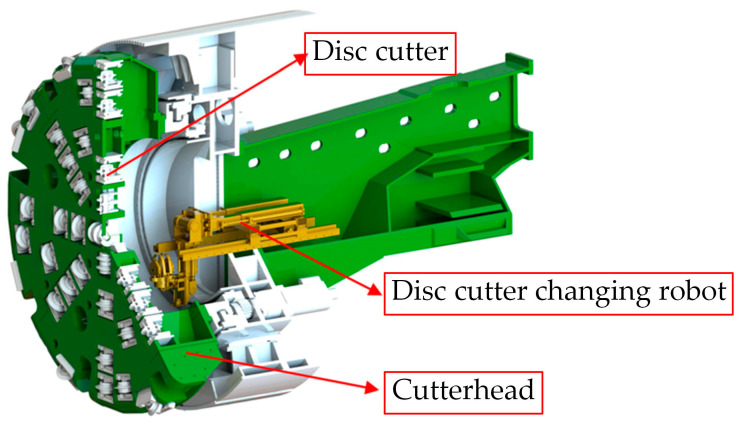
Operational stance of the shield disc cutter changing robot.

**Figure 2:**
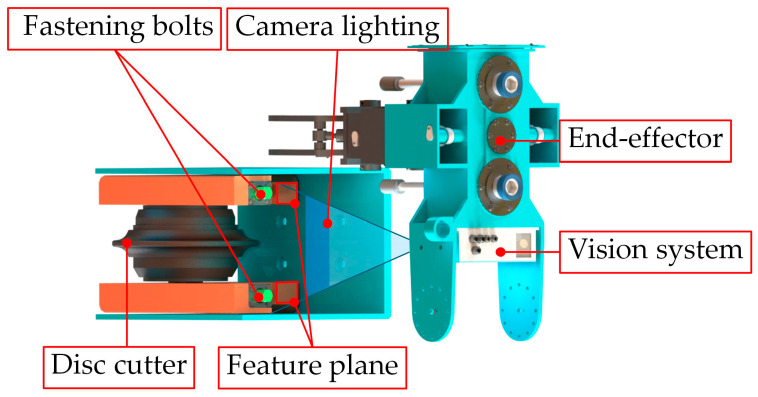
Localization of the cutterbox fastening system utilizing a vision-enabled end-effector.

During the automated disc cutter replacement process of the shield disc cutter changing robot, accurately locating the fastening bolts that secure the disc cutters within the cutterbox is essential for achieving complete automation. However, the effectiveness of the visual system in current shield disc cutter changing robots is limited by challenging underground conditions, such as restricted working space, low light levels, and contamination from mud and water. The operational position of the disc cutter changing robot is depicted in Figure 1, necessitating high positioning accuracy. To overcome these obstacles, an indirect visual localization method for the fastening bolts of the shield machine disc cutters has been developed, as shown in Figure 2. The precise localization of the bolts relies primarily on point cloud registration and instance segmentation of two-dimensional images.

Point cloud registration involves determining the pose transformation matrix that aligns multiple point clouds into a common coordinate system. This process facilitates the registration and integration of diverse point clouds. The advancement of point cloud registration methods primarily follows two approaches: optimization-based algorithms and deep learning-based algorithms.

Among the algorithms for optimization-based registration, Besl and McKay [5] introduced the Iterative Closest Point (ICP) algorithm. This method alternates between searching for correspondences and performing singular value decomposition (SVD) to derive the optimal transformation, thereby establishing a foundational approach for local point cloud registration methods. The Normal Distributions Transform (NDT) algorithm [6] segments the point cloud space into grids and employs Gaussian distributions to model the point distribution within each grid, facilitating probabilistic matching and registration of point clouds.

Recent advancements in deep learning have positioned point cloud registration methods as a significant area of research. The PointNetLK [7] algorithm utilizes deep learning techniques to estimate transformation parameters between point clouds. PCRNet [8] enhances this process by integrating PointNet [9] into the registration task, improving feature extraction and reducing the sensitivity of PointNetLK to noise. Huang et al. introduced the CAST method [10] for point cloud registration, which improves geometric consistency through the implementation of point-guided cross-attention and consistency-aware self-attention modules. This method, combined with a lightweight fine-matching module, effectively addresses the inefficiencies and inaccuracies associated with the lack of geometric consistency in coarse matching. Additionally, Pertigkiozoglou developed the BiEquiformer [11], a bidirectionally equivariant Transformer network that merges information across frames and layers, facilitating the extraction of high-quality superpoint correspondences and demonstrating significant robustness to variations in initial poses.

She et al. introduced PointDifformer [12], a robust method for point cloud registration that leverages graph neural partial differential equations (PDEs) and heat kernel features. This approach enhances noise resilience and registration accuracy by aggregating neighborhood information through a graph neural PDE module, incorporating heat kernel features into the attention mechanism, and utilizing a learnable singular value decomposition (SVD) module to predict transformations. Kang et al. developed the Equi-GSPR model [13], which integrates SE(3)-equivariant graph features within a graph neural network. By employing equivariant message passing, this model achieves rotation-invariant representation. It comprises a feature descriptor module, an equivariant graph layer, and a matching module, allowing it to effectively process sparsely sampled points while fully leveraging the intrinsic symmetry of point cloud data. Wang et al. [14] proposed an end-to-end registration method based on Transformers that incorporates dynamic positional encoding and triplet-angle positional encoding, integrates an enhanced cross-attention mechanism, and fuses multi-scale features to improve the accuracy and robustness of large-scale, low-overlap point cloud registration. In addition, several point–cloud and vision fusion methods [15] tailored for tunnel environments have been reported, providing valuable references for SLAM applications in tunneling engineering. However, their localization accuracy typically remains at the centimeter level, which is insufficient for the high-precision positioning required in the cutter-changing operations of the robot considered in this study.

In contrast to conventional correspondence-based techniques, deep learning methods extract neighborhood features on a point-by-point basis, greatly improving the resilience of point cloud registration to noise and occlusion. Nonetheless, these algorithms often entail substantial computational demands, which restrict their real-time applicability. Additionally, they exhibit a strong reliance on the overlap ratio between point clouds, indicating significant opportunities for further investigation and development in registration tasks that involve low-overlap or non-overlapping point clouds.

Instance segmentation not only classifies various object categories within an image but also differentiates between individual instances of the same category, allowing for precise recognition and segmentation of each object present. Notable examples include the YOLO series of algorithms and their subsequent enhancements. Wang et al. [16] tackled the difficulties associated with detecting water-horse obstacles in autonomous driving by introducing a lightweight, fine-grained instance segmentation model named LF-RTMDet. This model incorporates GhostConv, CARAFE, and PSA modules, leading to significant improvements in recognition accuracy and real-time performance, while also minimizing the number of parameters and computational complexity. It achieves real-time detection at 32 frames per second on the Jetson Orin Nano following quantization. Additionally, Akhtar developed an instance segmentation model called YOLOv11-SAMNet [17], which combines YOLOv11 and SAM for the automated detection and segmentation of 14 particle categories in urinary microscopic images, greatly enhancing the recognition accuracy of structures such as leukocytes and epithelial cells.

Qiu et al. [18] developed a lightweight segmentation model known as PS-YOLO-seg, which enhances feature extraction through structural compression and PSConv. This approach significantly reduces both computational complexity and model size while preserving high segmentation accuracy. Gu et al. [19] introduced a lightweight instance segmentation framework called PSC-YOLO, which enhances real-time perception accuracy and computational efficiency in urban settings by improving multi-scale feature learning and efficient mask decoding. Wang et al. [20] presented an efficient segmentation model named CS-YOLO, specifically designed for detecting concrete cracks. This model utilizes the StarConv structure, a lightweight segmentation head, and an NWD loss function based on the Wasserstein distance, enabling precise and rapid detection of fine cracks in complex backgrounds. Gao et al. [21] proposed a YOLO-based segmentation framework that integrates a lightweight GhostNet backbone for processing sonar images, along with a BiLSTM network for temporal feature learning. By predicting semantic vectors and bypassing computation layers for non-key frames, this framework enhances overall computational efficiency.

While existing point cloud registration methods in the literature, such as optimization-based ICP and deep learning-based approaches like Predator, demonstrate remarkable performance on point clouds with high overlap rates, they generally struggle with the extreme conditions encountered in shield tunnels. Specifically, when point clouds become extremely sparse and incomplete due to occlusions or sludge contamination, resulting in very low overlap, these direct registration methods often fail or suffer from severe accuracy degradation due to their inability to establish reliable point-to-point correspondences. The core objective of this work is to address this research gap: we propose an indirect image-point cloud fusion strategy, whose advantage lies in its independence from overlapping regions between point clouds. The method first extracts stable 2D feature planes from images through robust instance segmentation, then recovers camera poses via image feature matching, and finally infers bolt positions indirectly through coordinate transformation. This approach fundamentally circumvents the inherent challenges of low-overlap point cloud registration, thereby achieving millimeter-level positioning accuracy and exceptional stability even under severely degraded point cloud data. In Table 1 we show the comparison of other methods with the methods we propose.

This research tackles the difficulty of precisely locating fastening bolts of the shield disc cutter changing robot in extreme operating environments characterized by low illumination, slurry contamination, and limited working space. Direct localization of the bolts frequently results in incomplete point cloud data and imprecise positioning. To address this challenge, an indirect bolt indirect localization method approach is introduced, which identifies the bolt’s position by recognizing the rectangular feature surface adjacent to the disc cutters within the cutterbox, as illustrated in the feature plane of Figure 2.

The main contributions of this paper are summarized as follows:

(1) A novel SCMamba-YOLO model is introduced to facilitate efficient and precise segmentation of cutterbox feature surfaces.
(2) A non-overlapping point cloud registration method based on image-point cloud fusion is developed to tackle registration challenges posed by incomplete point clouds in demanding environments.
(3) An indirect bolt indirect localization method method based on feature surfaces is established, offering reliable coordinate inputs for the shield disc cutter changing robot.
(4) An experimental platform that simulates tunnel conditions is constructed to provide data support for the engineering application of the proposed methodology.

## 2. Materials and Methods

### 2.1. Indirect Localization Technique Utilizing Image and Point Cloud Integration

**Figure 3:**
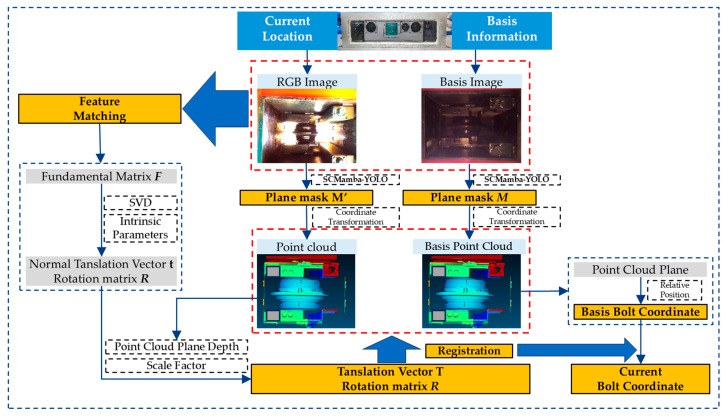
Schematic diagram of the proposed localization method.

The fundamental procedure of the proposed method is depicted in Figure 3. Initially, data from the reference image is gathered, which includes the coordinates of the corners and the bolt. Next, an image of the cutterbox in its current orientation is captured. This two-dimensional image is then processed through an instance segmentation model to produce mask information. By integrating the mask with the point cloud data, the point cloud corresponding to the feature plane is extracted, allowing for the determination of the corner coordinates.

Following this, feature matching is conducted between the reference image and the current RGB image to compute the rotation matrix. Utilizing the rotation matrix along with the corner information, the translation matrix is established. Ultimately, the pre-calculated coordinate transformation matrix facilitates the indirect localization of the bolt’s position.

### 2.2. Depthwise Separable Module-Based Image Segmentation Network with Multi-Level Feature Fusion

#### 2.2.1. Preliminaries

The state space model (SSM) [22] serves as a mathematical framework for representing the temporal progression of a dynamic system’s state. It delineates the system’s evolution over time using a collection of matrices and state variables. Typically, the model comprises a state equation and an output equation, and it can be analyzed in either continuous or discrete time. The mathematical formulation is defined as follows:

(1) $$h^{\prime }\left(t\right)\;=\;Ah\left(t\right)\;+\;Bx\left(t\right)$$

where $h^{\prime }\left(t\right)$ denotes the derivative of the current state *h*(*t*); $A\in R^{N\times N}$ represents the state transition matrix; $B\in R^{N\times N}$ is the input matrix; $C\in R^{N\times N}$ signifies the output matrix; and D indicates the command coefficient, which defines the direct impact of the input on the output. In the majority of state space models, *Dx*(*t*) = 0.

Mamba transforms the continuous parameters A and B into discrete parameters $\bar{A}$ and $\bar{B}$ using a predetermined discretization method, facilitating improved compatibility with deep learning frameworks. Following the application of Zero-Order Hold (ZOH) discretization, the state space model can be represented as follows:

(2) $$h_{k}\;=\;\bar{A}h_{k-1}{\;+\;\bar{B}x}_{k}$$

(3) $$y_{k}=Ch_{k}$$

where $\bar{A}=\exp\left(\Delta A\right)$ and $\bar{B}\;=\;(\Delta {A)}^{-1}\exp(\Delta A\;-\;I)\Delta B$, with Δ representing the time-scale parameter, *I* as the identity matrix, and *k* indicating the discrete time step. It can be noted that the discretized state space models (SSMs) exhibit a structural resemblance to recurrent neural networks (RNNs).

By constructing a convolution kernel $\bar{K}\;=\;(C\bar{B},C\bar{AB},\cdots {,C\bar{A}}^{L-1}\bar{B}$, the entire sequence transformation can also be represented in a convolutional form as follows:

(4) $$y\;=\;x\;\times \;\bar{K}$$

#### 2.2.2. Overall Architecture

This study introduces an image segmentation network named SCMamba-YOLO, which incorporates the OS2 Block module along with multi-level feature fusion techniques.

**Figure 4:**
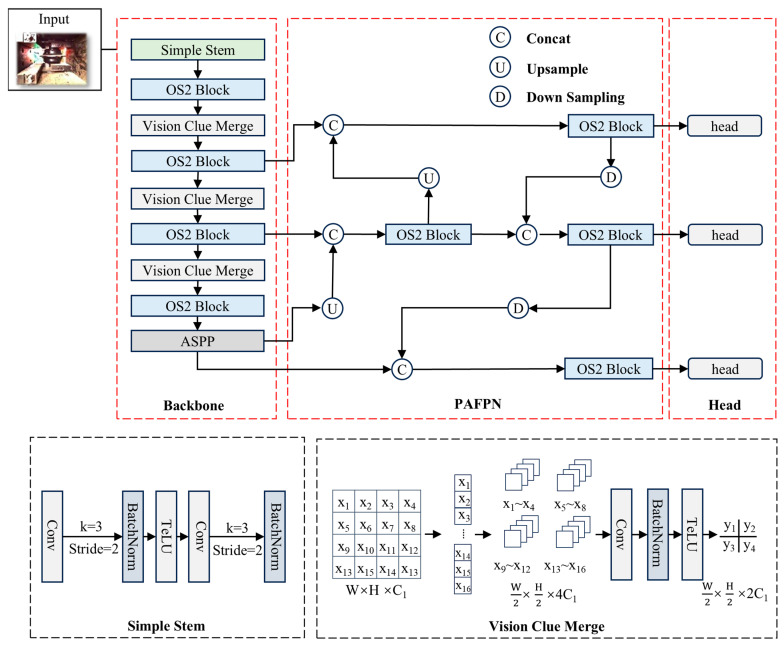
Architecture of the SCMamba-YOLO model.

The architecture of SCMamba-YOLO is depicted in Figure 4 and comprises three primary components: the Backbone, PAFPN [23], and Head. Initially, the input image is processed through the Simple Stem module, which facilitates rapid downsampling and preliminary feature encoding. Following this, the Backbone integrates multiple layers of OS2 Blocks and Vision Clue Merge modules in an alternating fashion, culminating in the introduction of an ASPP [24] module designed to expand the receptive field. This enhancement improves multi-scale semantic representation while preserving computational efficiency. The PAFPN neck employs a bidirectional feature fusion approach, allowing for effective interaction between high- and low-level features through multi-level feature concatenation and upsampling. To refine contextual information, DSBlock modules and lightweight convolutions are strategically placed along critical pathways. The Head section is composed of three detection branches that generate predictions at various scales, thereby ensuring precise detection of small, medium, and large objects.

#### 2.2.3. OS2 Block

Following preprocessing with the Simple Stem module, the feature map undergoes deep feature extraction through a sequence of OS2 Blocks. Let us denote the input feature $Z^{l-3}$ as having dimensions of $R^{C\times H\times W}$, which can be represented as follows:

(5) $$Z^{l-2}\;=\;\sigma (\mathrm{BatchNorm}\left(\mathrm{ConvModule}\left(Z^{l-3}\right)\right))$$

where σ represents the TELU activation function [25].

(6) $$Z^{l-1}=\mathrm{SS}2D(\mathrm{LayerNorm}\left(Z^{l-2}\right){\;+\;\mathrm{SCSA}(Z}^{l-2})$$

(7) $${Z\;}^{l}=\mathrm{RGBlock}\left(\mathrm{LayerNorm}\left(Z^{l-1}\right)\right){\;+\;\mathrm{SCSA}(Z}^{l-1})$$

**Figure 5:**
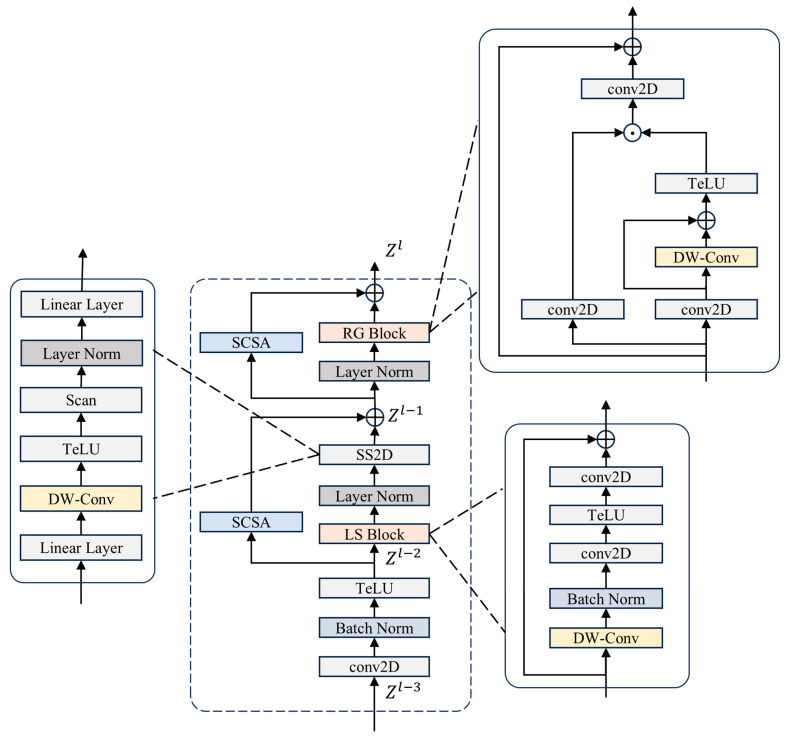
Structure of the OS2 Block module.

The architecture of the OS2 Block module is illustrated in Figure 5. The input feature map undergoes processing by the LS Block and SS2D modules, which aim to improve local spatial features while integrating global relationships via multi-directional scanning. To reinforce essential information across both channel and spatial dimensions, Selective Cross-Spatial Attention (SCSA) [26] is applied both prior to and following the SS2D module. Finally, the RG Block employs a residual gated structure that maintains global contextual information and enhances feature representation through nonlinear transformations.

The LS Block is primarily composed of convolutional layers, normalization layers, nonlinear activation functions, and residual connections. This architecture significantly lowers computational complexity while preserving robust feature representation capabilities, facilitating stable training and rapid convergence in deep neural networks.

SS2D [27] effectively integrates global dependency modeling with local spatial feature extraction, greatly improving the ability to represent features while ensuring low computational complexity.

**Figure 6:**
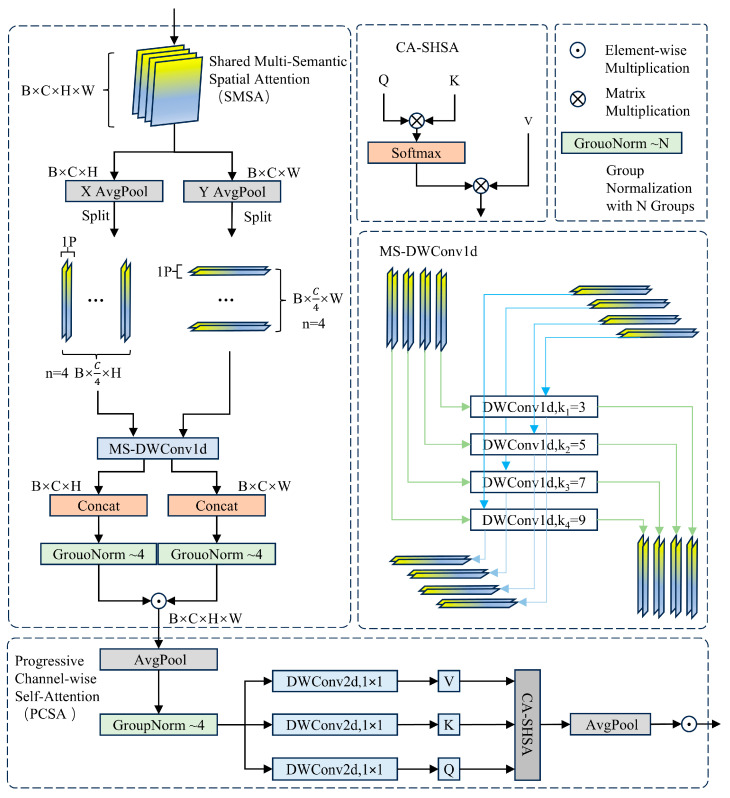
Structure of the SCSA mechanism.

The architecture of the SCSA mechanism is depicted in Figure 6. The SCSA module comprises two key elements: Shared Multi-Semantic Spatial Attention (SMSA) and Progressive Channel-wise Self-Attention (PCSA). This configuration enhances feature interaction through the self-attention mechanism in PCSA, which effectively mitigates the discrepancies in multi-semantic information across various sub-features in SMSA.

The SMSA utilizes multi-scale depthwise 1D convolutions to capture spatial information across various semantic levels from four distinct sub-features. This approach facilitates the integration of multi-semantic information and, through a progressive compression strategy, infuses discriminative spatial priors into the channel self-attention mechanism of PCSA, thereby effectively accomplishing channel recalibration.

PCSA integrates an advanced compression approach with a channel-specific self-attention mechanism (CSA) to reduce computational complexity while maintaining the spatial priors acquired in SMSA. Furthermore, PCSA utilizes the self-attention mechanism to delve deeper into channel-level similarities, effectively diminishing semantic inconsistencies among various sub-features.

The RG Block utilizes a conventional residual group architecture that standardizes feature values to enhance training stability. It retains a high capacity for representation while also being lightweight, effectively capturing intricate local patterns and alleviating gradient degradation challenges in deep neural networks.

### 2.3. Transformation of Point Cloud to RGB Image Plane Projection

**Figure 7:**
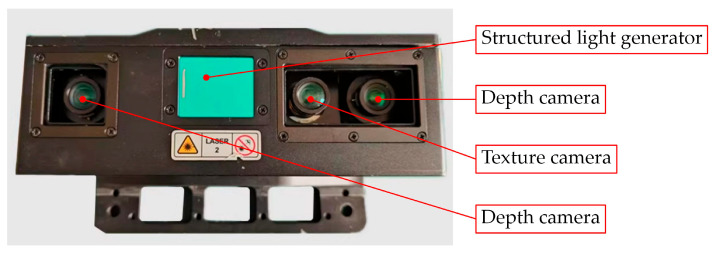
Positions of lenses in industrial cameras.

The vision system integrated into the end-effector comprises a texture camera and a depth camera, which are spatially positioned at different angles. As a result, the data obtained from these two cameras are represented in distinct coordinate systems, as depicted in Figure 7. During the data processing phase, it is essential to align the point cloud generated by the depth camera with the RGB image captured by the texture camera. This alignment allows for the application of the RGB image mask, segmented by the SCMamba-YOLO model, onto the point cloud data. To facilitate this process, it is crucial to establish a transformation relationship between the two coordinate systems.

The obtained point cloud is initially uniformly downsampled to decrease its density, thereby removing any potential non-uniform distributions present in the raw data while maintaining its geometric characteristics. The resulting data is referred to as the input point cloud Pdepth. Utilizing the calibration software Mech-Eye Viewer 2.4.0 provided by the manufacturer, the intrinsic parameters for both the texture and depth cameras are determined. This includes the principal point and focal length matrices, denoted as $K_{texture\;\;Camera}$ and $K_{depth\;\;Camera}$, along with the rotation matrix *R_camera_* and the translation matrix *t_camera_*, which represent the spatial relationship between the two cameras.

The matrix containing the principal point and focal length information for the texture camera is represented as indicated in Equation (8).

(8) $$K_{\mathrm{textureCamera}}=\left[\begin{array}{ccc} f_{x} & 0 & c_{x}\\ 0 & f_{y} & c_{y}\\ 0 & 0 & 1 \end{array}\right]$$

In this context, *f_x_* and *f_y_* indicate the focal lengths of the camera along the *x* and *y* axes, respectively. Meanwhile, *c_x_* and *c_y_* refer to the coordinates of the principal point, which represent the intersection of the optical axis with the image plane in the *x* and *y* dimensions.

The conversion between the coordinate systems of the texture camera and the depth camera is characterized by the rotation matrix *R_camera_* and the translation matrix *t*_camera_. The input point cloud *P_depth_* is transformed from the depth camera’s coordinate system to that of the texture camera via rotation and translation, yielding the aligned point cloud *P_texture_*, as illustrated in Equation (9).

(9) $$P_{\mathrm{texture}}{=R}_{\mathrm{camera}}{\cdot P}_{\mathrm{depth}}^{T}{\;+\;t}_{\mathrm{camera}}$$

Under the aforementioned conditions, the conversion from the depth camera coordinate system to the texture camera coordinate system can be achieved. By utilizing perspective projection alongside the intrinsic parameter matrix of the texture camera, the three-dimensional coordinates *P_texture_*(*X_t_*,*Y_t_*,*Z_t_*) are transformed into two-dimensional coordinates *p*(*u*,*v*), which are ultimately aligned with the RGB image coordinate system. The detailed projection equation is presented in Equation (10).

(10) $$\left\{\begin{array}{c} u=\frac{f_{x}X_{t}}{Z_{c}}{+c}_{x}\\ v=\frac{f_{y}Y_{t}}{Z_{c}}{+c}_{y} \end{array}\right.$$

By utilizing the aforementioned transformation relationship, the point cloud is mapped into the RGB image coordinate system, thereby achieving coordinate registration.

### 2.4. Extraction of Corner Coordinates Through the Integration of Image and Point Cloud Data

**Figure 8:**
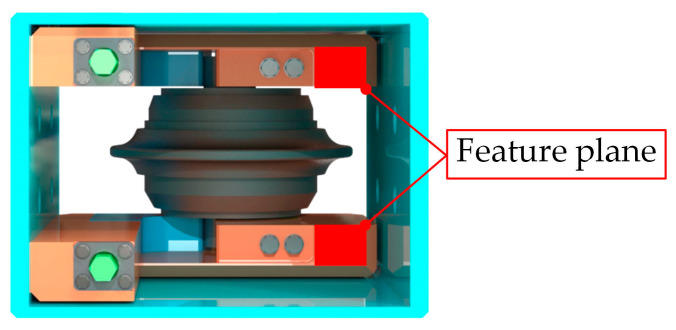
Mask of the feature plane produced by the SCMamba-YOLO model.

Upon capturing a new cutterbox image, the SCMamba-YOLO instance segmentation model autonomously detects the feature plane and produces the corresponding mask, as shown in Figure 8.

Simultaneously, the mask information, which encompasses the bounding box b, is produced. 

Utilizing the generated mask data, segmentation is conducted on the projected point cloud image, preserving only the 2D point cloud data located within the masked area. The corner coordinate information is subsequently extracted from this retained segment during further processing.

Point cloud reconstruction mainly involves two key elements: the discrete mapping of two-dimensional image coordinates and the inverse projection of depth data.

The process of mapping 2D image coordinates to depth values is discretized. A two-channel accumulation buffer *M_buffer_∈R^H^*^×*W*×2^ is created: Channel 0 accumulates depth values corresponding to the image coordinates *p*^(*i*)^, while Channel 1 records the number of valid samples for each coordinate. For each texture coordinate *p*^(*i*)^, invalid regions are filtered out using a mask, retaining only the point cloud data within the masked rectangular Region of Interest (ROI). Within the ROI, the data of *p*^(*i*)^ is distributed to its four neighboring pixels $u_{i}+\delta_{u},v_{i}+\delta_{v},\forall \delta_{u},\delta_{v}\in \left\{0,1\right\}$. For each valid pixel within the mask, the corresponding accumulation operation is performed in the buffer, as described in Equation (11).

(11) $$\left\{\begin{array}{c} M_{\mathrm{buffer}}{[v+\delta }_{v}{,u+\delta }_{u}{,0]+d}_{i}\\ M_{\mathrm{buffer}}{[v+\delta }_{v}{,u+\delta }_{u},1]+1 \end{array}\right.$$

In the equation, *d_i_* denotes the depth value of the current pixel. This processing method aids in reducing quantization errors resulting from coordinate discretization and improves the continuity of the depth map.

(1) Depth inverse projection utilizing the camera model: For every pixel (*x*,*y*) located within the region of interest (ROI), the average depth *d* is determined as outlined in Equation (12).

(12) $$d=\frac{M_{\mathrm{buffer}}\left(x,y,0\right)}{M_{\mathrm{buffer}}\left(x,y,1\right)}$$

(2) The inverse projection equation is utilized to reconstruct 3D points, resulting in the generation of the point cloud ***P_reconstruct_*** within the texture camera coordinate system, as indicated in Equation (13).

(13) $$P_{\mathrm{reconstruct}}=\left[\begin{array}{c} X_{r}\\ Y_{r}\\ Z_{r} \end{array}\right]=\left[\begin{array}{c} \frac{d\cdot (u-c_{x})}{f_{x}}\\ \begin{array}{c} \frac{d\cdot (v-c_{y})}{f_{y}}\\ d \end{array} \end{array}\right]$$

We utilize the SCMamba-YOLO instance segmentation model to refine the point cloud *P*_reconstruct_ within the region of interest (ROI).

An objective function is formulated to identify corner coordinates characterized by significant segmented features, as illustrated in Equation (14).

(14) $$min\;{f(p}^{\left(i\right)}{)=|x}_{i}{|+|y}_{i}|$$

By optimizing this function, the points within the point cloud that project onto the XOY plane with the least total absolute axis distances are identified. The coordinates of the two points nearest to the origin, characterized by the smallest sum of absolute X and Y values, are designated as corner coordinates Pcornerpoint_k and are retained for the subsequent calculation of the translation matrix during the coordinate transformation process.

### 2.5. Method for Registering Non-Overlapping Point Clouds

The bolt coordinates in the reference image can be obtained for the current image using a rigid-body coordinate transformation, which involves calculating rotation and translation matrices. In accordance with this approach, the rotation matrix *R* and the translation matrix t are established through a feature-matching process. The reference image acts as the baseline image containing known bolt coordinate data. Given the challenging conditions in tunnel environments, such as scale variations due to changing distances, viewpoint rotations caused by camera alignment, and low illumination levels, a robust feature matching algorithm is essential. SIFT algorithm is utilized to facilitate feature matching between the two images due to its strong performance in scale invariance, rotation invariance, and stability under illumination changes. SIFT achieves this by constructing a scale space to handle size variations, detecting keypoints using the Difference of Gaussian (DoG) function, and assigning orientations to keypoints to ensure rotation invariance. Additionally, SIFT relies on gradient-based descriptors, which are less sensitive to absolute brightness variations, making it suitable for low-light conditions commonly encountered in tunnel settings.

Utilizing the previously acquired SIFT matched points and the intrinsic parameters of the camera, the geometric relationship of a point across two images can be articulated through the essential matrix. This matrix facilitates the recovery of the camera pose of the reference image based on the current camera pose. A 3D point, along with its projections p1 and p2 in the two cameras, is interconnected via the extrinsic matrices *R* and *t*, resulting in an equation that embodies the epipolar constraint.

**Figure 9:**
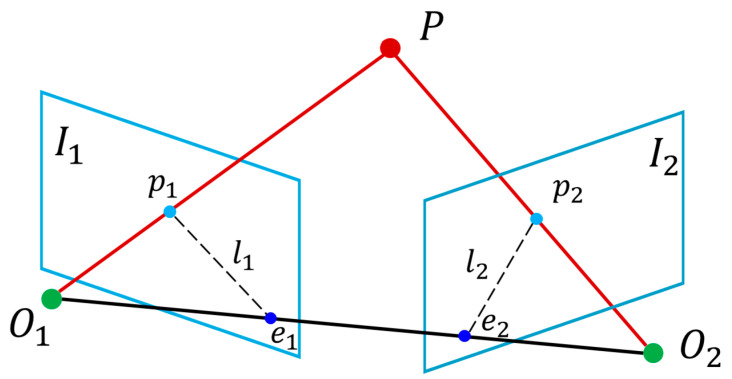
Schematic diagram of the epipolar constraint principle.

Epipolar geometry illustrates the relationship between the projections of a three-dimensional point across two different views. Specifically, if a spatial point *P* is projected onto the image plane *I*_1_ of camera *O*_1_ as *p*_1_, and onto the image plane *I*_2_ of camera *O*_2_ as *p*_2_, then *p*_2_ is constrained to lie on the epipolar line *l*_2_ associated with *p*_1_, as depicted in Figure 9.

The essential matrix *E* serves as the mathematical embodiment of this constraint, fundamentally articulated through the epipolar constraint, as illustrated in Equation (15).

(15) p2Ep2T=0

In this context, *p*_1_ and *p*_2_ represent the homogeneous coordinates of spatial points within the normalized image coordinate system. To derive normalized coordinates, it is essential to decouple the acquired matching points from the camera’s intrinsic parameters *K*. This process facilitates the determination of the rigid motion relationship between the two camera coordinate systems, independent of the intrinsic parameters. The detailed calculation formula is presented in Equation (16).

(16) $$X=K^{-1}\left[\begin{array}{c} u\\ v\\ 1 \end{array}\right]$$

In the normalized coordinate system, the essential matrix *E* is defined, and its computation is presented in Equation (17).

(17) E=t × R

The essential matrix *E* can be decomposed into the rotation matrix *R* and the translation vector *t*.

Subsequently, the eight-point algorithm is employed to derive a linear solution for the essential matrix. For every pair of corresponding points $\left(x_{1}^{\left(i\right)},x_{2}^{\left(i\right)}\right)$, the equation can be elaborated as demonstrated in Equation (18).

(18) $$\left[\begin{array}{ccc} x_{2}^{\left(i\right)}x_{1}^{\left(i\right)} & x_{2}^{\left(i\right)}y_{1}^{\left(i\right)} & x_{2}^{\left(i\right)}\\ y_{2}^{\left(i\right)}x_{1}^{\left(i\right)} & y_{2}^{\left(i\right)}y_{1}^{\left(i\right)} & y_{2}^{\left(i\right)}\\ x_{1}^{\left(i\right)} & y_{1}^{\left(i\right)} & 1 \end{array}\right]\cdot \mathrm{vec}(E)=0$$

In the equation, *vec*(*E*) denotes the vectorized representation of ***E***.

A linear equation system *A*e = 0 is formulated using a minimum of eight points. Applying singular value decomposition (SVD) to the essential matrix facilitates the retrieval of the camera motion parameters (***R***,***t***). As indicated in Equation (19), there are four potential solutions available.

(19) $$\left\{\begin{array}{cc} R_{1}{=UR}_{z}\left(+\pi /2\right)V^{T}, & t_{1}{=Ue}_{3}\\ R_{2}{=UR}_{z}(-{\pi /2)V}^{T}, & t_{2\;}{=Ue}_{3}\\ R_{3}{=UR}_{z}\left(+\pi /2\right)V^{T}, & t_{3}=-{Ue}_{3}\\ R_{4\;}{=UR}_{z}(-{\pi /2)V}^{T}, & t_{4}=-{Ue}_{3} \end{array}\right.$$

The formula for calculating $R_{z}(\theta )$ is presented in Equation (20), with the condition that **e**_3_ = ${\left[0,0,1\right]}^{T}$.

(20) $$R_{z}(\theta )=\left[\begin{array}{ccc} \cos\theta & -\sin\theta & 0\\ \sin\theta & \cos\theta & 0\\ 0 & 0 & 1 \end{array}\right]$$

A positive-negative depth consistency check is conducted on the 3D points, and the solution that maximizes the number of points for which ***Z*** > 0 is chosen as the final outcome, resulting in the desired rotation (***R***) and translation (***t***).

In practical applications, the quantity of matched keypoints usually exceeds eight pairs. Given that only eight pairs are utilized for a single computation of the essential matrix, the resulting confidence may be diminished. Consequently, the additional points can serve as validation points to evaluate the reliability of the estimated essential matrix. Integrating this approach with the RANSAC estimation algorithm can substantially enhance both the confidence and robustness of the essential matrix.

Initially, eight pairs of corresponding points are chosen from the correspondence set to formulate a linear equation system for the computation of the essential matrix. Following the acquisition of the essential matrix, the error εi for each point pair is computed using Equation (21), which is subsequently utilized to identify the inliers.

(21) $$\varepsilon_{i}=\frac{\left|x_{2}^{(i)T}E_{k}x_{1}^{\left(i\right)}\right|}{\sqrt{{{(E}_{k}x_{1}^{\left(i\right)})}^{2}{+(E}_{k}x_{2}^{\left(i\right)}{)}^{2}}}$$

**Figure 10:**
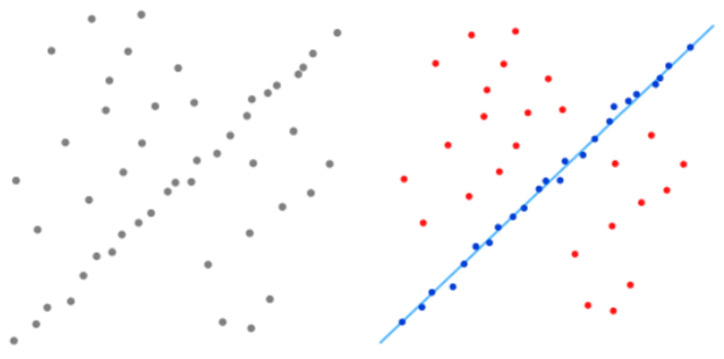
The optimal line derived from the point set utilizing the RANSAC algorithm.

A point pair is classified as an inlier when $\varepsilon_{i}<\tau$, as demonstrated in Figure 10.

Subsequent iterations are conducted, with the total number of iterations *M* being dictated by the confidence level *q* and the outlier ratio *e*. The precise calculation formula is presented in Equation (22).

(22) $$M=\frac{\log(1-q)}{\log(1-{(1-e)}^{5})}$$

The error threshold τ is closely associated with the pixel reprojection error, with τ=1.0 indicating an approximate tolerance of one pixel. By following the computation process outlined above, the optimal essential matrix can be derived, allowing for the identification of the final inliers.

### 2.6. Acquisition of Coordinates for Fastening Bolts

Due to the absence of depth information in a 2D image, the rotation matrix R serves as the sole determinant of the true rotational scale, while the translation vector t indicates direction without providing any scale information. The corner coordinates of the image currently being processed are transformed using the rotation matrix R to derive the rotated corner coordinates. The deviation in coordinates between these transformed points and the standard image corners reflects only a translational offset. By calculating the difference between the two sets of coordinates, a translation vector with real-world scale can be determined. Two corner coordinates from the standard image, designated as P245_0 and P245_1, are chosen as reference points.

The translational adjustment is calculated based on Equation (23).

(23) $${\Delta t}_{k\;}{=p}_{\mathrm{corner}\_k}-p_{245\_k}\left(k=0,1\right)$$

The final translation matrix is determined by averaging the two calculated correction vectors, referred to as Δt. The ultimate coordinates of the bolts are calculated according to Equation (24).

(24) $$p_{\mathrm{current}\;}{=R\cdot p}_{i}^{245}+\Delta t$$

Consequently, the final coordinates of the bolts are calculated.

## 3. Experiments, Results, and Analysis

### 3.1. Instance Segmentation Experiments

#### 3.1.1. Datasets

**Figure 11:**
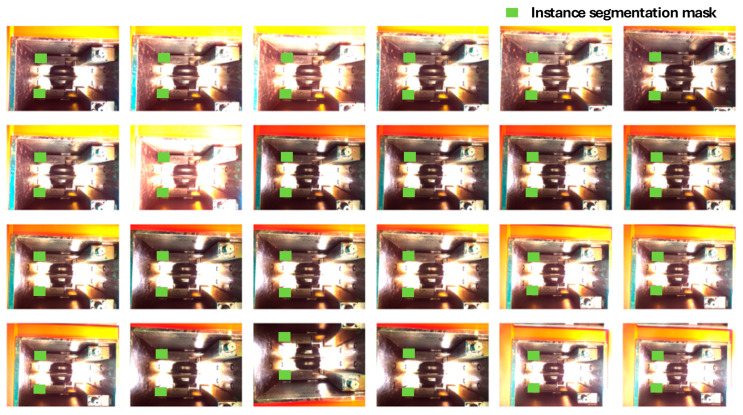
The developed disc cutter box dataset, referred to as HCBDataset, where the green squares in the figures indicate the locations of the instance segmentation masks.

We developed a novel instance segmentation dataset comprising 200 images, referred to as HCBDataset. This dataset features well-lit and clear images of disc cutter boxes, as well as poorly illuminated images and complex scenes with significant background interference, the specific distribution is shown in the following Table 2. It should be specially noted that all images were captured from a frontal view, which is achieved by precisely adjusting the robot’s pose. This ensures the camera is perpendicularly aligned with the cutterbox feature planes corresponding to side disc cutters, upper disc cutters, lower disc cutters, and other positions, thereby eliminating the interference caused by viewpoint deviations. The varied acquisition conditions contribute to the model’s robustness and generalization capabilities in instance segmentation tasks. To ensure precise annotations, we utilized the Labelme tool for pixel-level instance labeling of the disc cutters box feature surfaces within the images, exporting the segmentation masks in COCO format. All targets were categorized under a single label, “disc cutters box feature plane.” Several annotated examples are illustrated in Figure 11, where the colored masks represent the instance segmentation labels. Following annotation, the dataset was further processed into a segmentation format that is directly compatible with SCMamba-YOLO for model training.

#### 3.1.2. Implementation Details

A robust and efficient hardware platform is essential for model training. In this research, experiments were performed on a DELL Precision T7920 workstation (Dell Inc., Round Rock, TX, USA), which features an Intel Xeon E5-2698v3 processor (Intel Corporation, Santa Clara, CA, USA), dual Tesla V100 GPUs (16 GB each), and 64 GB of RAM. The software environment utilized included Ubuntu 22.04, Anaconda3, CUDA 12.1, and Python 3.9, with PyTorch 2.2.2 employed as the deep learning framework, ensuring reliable support for the experimental procedures.

#### 3.1.3. Comparison with Primary Methods

To thoroughly assess the proposed model, SCMamba-YOLO was evaluated against five prominent instance segmentation techniques on the HCBDataset, specifically YOLOv11-n-segment, RTMDet-Ins-T, RTMDet-Ins-S, YOLOv11-s-segment, and SparseInst-R50. As illustrated in Table 3, the bold values indicate the highest performance metrics, while “–” signifies that the relevant data were either not provided in the respective publications or that pretrained weights were unavailable to the public. The comparison results indicate that SCMamba-YOLO surpasses both YOLOv11-n-segment (mAP50 = 86.2%, mAP50-95 = 76.8%) and YOLOv11-s-segment (mAP50 = 88.2%, mAP50-95 = 79.6%) in segmentation accuracy, achieving mAP50 = 90.7% and mAP50-95 = 82.2%. Additionally, when compared to RTMDet-Ins-T, RTMDet-Ins-S, and SparseInst-R50, the proposed model demonstrates superior computational efficiency, exhibiting lower FLOPs (13.36 G) and reduced latency (1.51 ms). Moreover, SCMamba-YOLO maintains a relatively modest parameter count (5.92 M), reflecting an effective balance between accuracy and computational expense. These findings substantiate the exceptional performance of SCMamba-YOLO on the HCBDataset, highlighting its advantages in both precision and efficiency.

#### 3.1.4. Ablation Experiment

To investigate the contribution of the proposed improvements, a series of ablation experiments were performed using YOLO as the baseline model, as shown in Table 4 below. The analysis focused on the performance enhancement yielded by the ASPP module, OS2 Block (with/without SCSA), and their combinations.

Table 3 presents the ablation results at IoU = 0.5, demonstrating that integrating these components improves both mAP50 and mAP50-95 to varying degrees. The individual addition of the ASPP module (Group 2) boosts mAP50 from 0.864 to 0.871 (+0.7%) and mAP50-95 from 0.771 to 0.793 (+2.2%). Incorporating the OS2 Block without SCSA (Group 3) increases mAP50 to 0.883 (+1.9%) and mAP50-95 to 0.802 (+3.1%). Using the OS2 Block with SCSA alone (Group 4) elevates mAP50 to 0.894 (+3.0%) and mAP50-95 to 0.809 (+3.8%), indicating that SCSA effectively enhances feature interaction and further improves segmentation performance.

For the combined schemes, jointly integrating the ASPP module and OS2 Block without SCSA (Group 5) raises mAP50 to 0.887 (+2.3%) and mAP50-95 to 0.804 (+3.3%). The combination of the ASPP module and OS2 Block with SCSA (Group 6) achieves the highest performance, improving mAP50 from 86.4% to 0.907 (a 4.3% increase) and mAP50-95 from 0.771 to 0.822 (a 5.1% increase) over the baseline Mamba-YOLO-T network. This confirms the synergistic effect of the ASPP module (expanding receptive field) and OS2 Block with SCSA (strengthening feature representation), which significantly improves detection accuracy and generalization ability of the model.

### 3.2. Experiments in Ideal Conditions

To assess the viability of the proposed visual localization method, an experiment was conducted to validate localization accuracy using an integrated laboratory disc cutter system. Several repeated trials were executed to determine the coordinates of the bolts and to compare these measurements with the established ground truth values.

**Figure 12:**
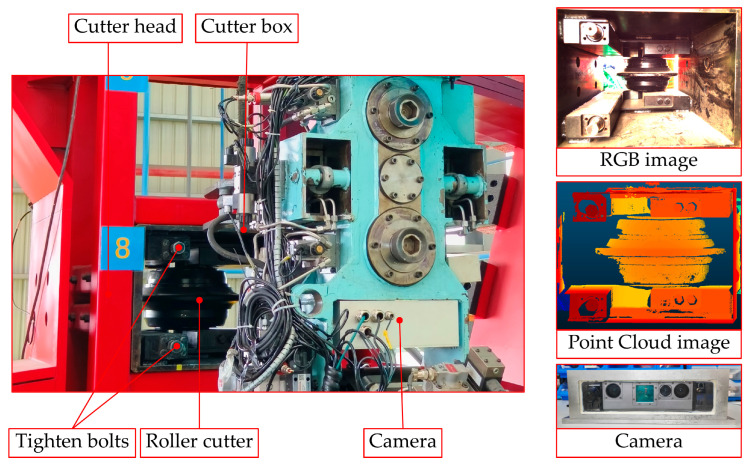
An end-effector integrated with a visual system for bolt indirect localization method in a laboratory setting.

Figure 12 depicts the experimental arrangement used to verify localization accuracy, which comprises the tool holder, disc cutter box, 19-inch disc cutters, LED lighting, industrial camera, and the camera mounting stand. The detailed specifics of the experiment are outlined as follows:

(1) Image Acquisition: An industrial camera was employed to capture images, achieving a depth image resolution of 2048 × 1536 pixels and a texture image resolution of 2000 × 1500 pixels.
(2) Distance: The distance from the camera stand to the tool holder was set at 600 mm
(3) Lighting: Illumination was provided by an LED light source.
(4) Targets: Two fastening bolts located at key positions within the disc cutter box were designated as target objects.

**Figure 13:**
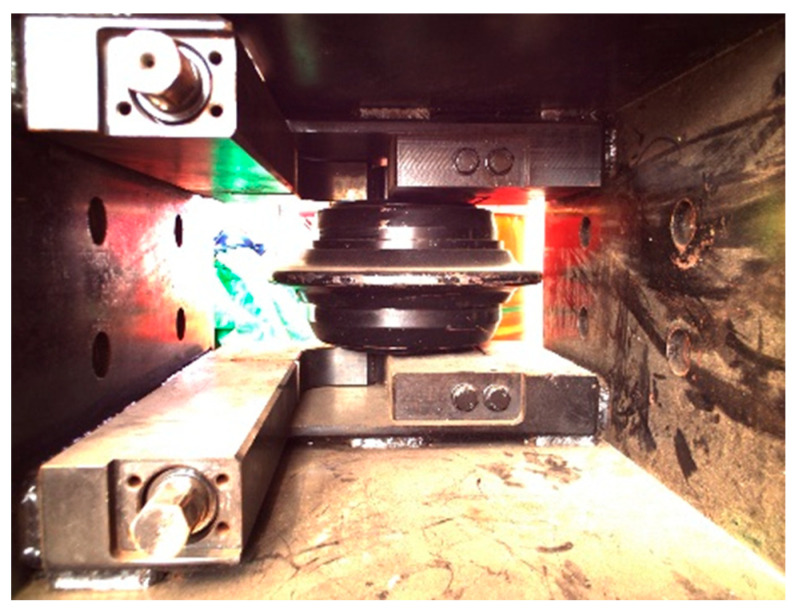
Photograph of the disc cutter housing taken in a controlled laboratory environment.

In the course of the experiments, the camera was oriented towards the bolt area of the disc cutter box in well-illuminated conditions, with an imaging distance of approximately 600 mmA total of six sets of images were collected, each comprising both texture and depth images. The RGB image depicting the interior of the disc cutter box obtained during the experimental setup is presented in Figure 13.

Experimental Results and Analysis: The localization error results for each image group are presented in the Table 5 below. Among the 12 samples analyzed, the absolute error across all axes was ≤1.98 mm, which is below the acceptable tolerance level (≤3 mm) for bolt indirect localization method in industrial tool systems. This finding confirms that the proposed visual localization method achieves adequate accuracy under optimal laboratory conditions. The mean absolute error along the Z-axis (axial depth) was measured at only 0.32 mm, with 11 out of 12 samples exhibiting errors ≤ 1 mm, indicating excellent axial localization precision. Throughout all six experimental groups, no significant deviations were noted, demonstrating strong repeatability and stability. This verifies that the proposed approach can be reliably utilized for bolt indirect localization method tasks in real-world applications.

### 3.3. Experimental Platform for Tunnel Environments

To further assess the stability of the proposed visual localization technique, an orthogonal accuracy verification experiment was conducted within an integrated disc cutter system that simulated a real tunnel environment. This simulation aimed to evaluate the method’s robustness and algorithmic adaptability in the presence of common on-site interferences, including viewpoint deviation, illumination changes, and mud-water contamination, thereby providing essential data for subsequent engineering applications. Various influencing factors, such as illumination fluctuations, mud adhesion, and the distance between the camera and target, were introduced to test the reliability of the visual system. The performance of three feature matching algorithms—SIFT (the algorithm utilized in this study), AKAZE, and ORB—was compared, and accuracy verification experiments were performed under the aforementioned environmental conditions.

In addition to the current experimental setup, supplementary equipment including light-blocking fabric, a mud-water mixture, a measuring tape, and a high-pressure water jet was added. The specifics of these additions are detailed below.

(1) Adjustment of the camera-to-cutterbox angle: Three imaging configurations were evaluated—(a) camera positioned off-axis without direct alignment to the cutterbox, (b) camera aligned directly with the cutterbox, and (c) camera rotated at an angle within the shooting perspective.
(2) Illumination simulation: Various lighting intensities and light-blocking materials were employed to replicate low-light tunnel environments, encompassing three illumination conditions—(d) absence of auxiliary lighting, (e) minimal auxiliary lighting, and (f) enhanced auxiliary lighting (normal control group).
(3) Simulation of mud-water contamination: Different levels of mud adhesion were applied to mimic tunnel pollution scenarios, classified as (g) light mud-water adhesion, (h) moderate sediment adhesion, and (i) heavy sediment adhesion.
(4) Variation in camera distance: The distance from the camera to the cutterbox was modified across three levels—(j) close (300 mm), (k) medium (600 mm), and (l) distant (900 mm).
(5) Comparison of algorithms: The experimental data were analyzed using three feature matching algorithms—SIFT, AKAZE, and ORB—to determine the coordinates of the bolts.

**Figure 14:**
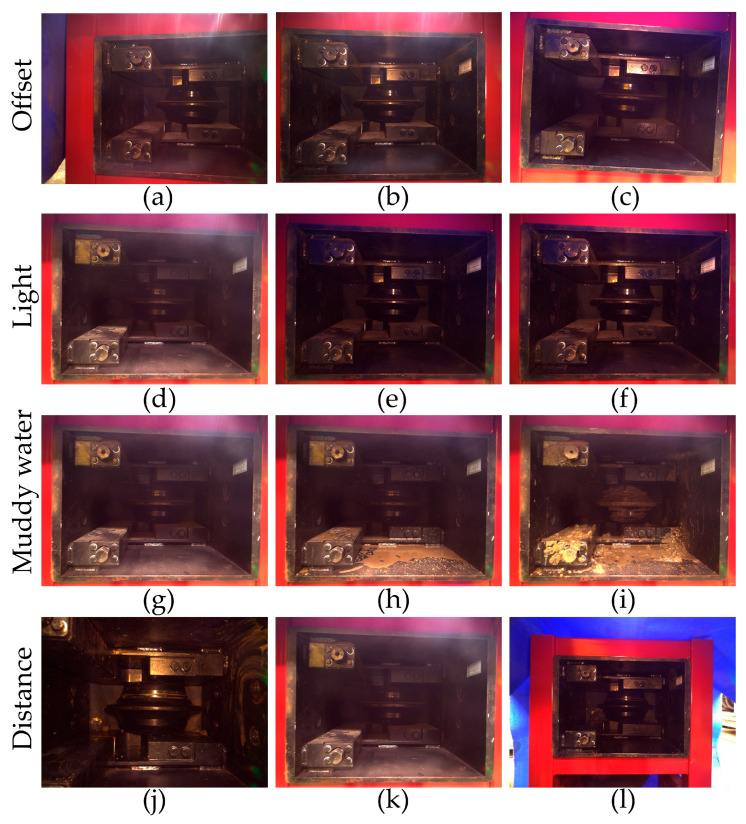
Images of cutterboxes obtained in simulated tunnel environments.

Experimental scenarios were established under various operational conditions utilizing illumination simulation equipment, which included adjustable light sources and light-shielding fabric. Different levels of contamination were manually simulated using mud and a washing water jet, while measurement tools and adjustable supports were employed to regulate the data acquisition distance. The RGB images of the interior of the cutterbox, captured by the texture camera under these experimental conditions, are presented in Figure 14. Three prominent feature matching algorithms—SIFT, AKAZE, and ORB—were utilized to analyze the texture images obtained from each working condition. The 3D coordinates of the bolts were calculated and compared with the ground truth values to assess the robustness of each algorithm against interference.

Experimental Results: Table 6 displays the error metrics for each image set processed using the SIFT algorithm, while Table 7 outlines the corresponding results for the AKAZE and ORB algorithms. Table 8 presents the feature matching results of different algorithms under the aforementioned environments. The SIFT-based localization method demonstrates excellent adaptability to variations in viewpoint, robust performance under changes in illumination, and consistent results across different distances. In contrast, the AKAZE method shows considerable localization errors in 50% of the coordinate estimations, proving reliable only under mild disturbances, with accuracy significantly declining in more complex environments. The ORB method performs the least effectively, with 66.6% of the localization outcomes exhibiting substantial errors, highlighting its inadequate robustness in challenging tunnel conditions. These findings validate that the proposed SIFT-based bolt indirect localization method approach achieves high localization accuracy and exhibits strong adaptability to environmental variations.

### 3.4. Assessment of the Efficacy of the Non-Overlapping Point Cloud Registration Technique

The experiments carried out in both ideal and tunnel environments demonstrated that the SIFT-based visual localization method achieves high accuracy and robust performance in bolt indirect localization method. However, the adaptability of various mainstream point cloud registration methods to non-overlapping point cloud scenarios within an integrated disc cutter system remains unclear. To address this, a comparative experiment was conducted to assess the localization accuracy of several mainstream point cloud registration methods in relation to the proposed approach under tunnel conditions. Four representative registration methods were selected for comparison: global geometric feature-based (FPFH), Iterative Closest Point (ICP), a deep learning-based method (Predator), and the SIFT-based method introduced in this study.

Among the various methods, Fast Point Feature Histograms (FPFH) demonstrates limited adaptability due to its strong reliance on the geometric integrity of the point cloud. In scenarios where mud obstructs visibility (e.g., scenario i), the absence of geometric features hampers the FPFH histogram’s ability to accurately represent the bolt structure, resulting in frequent mismatches. Additionally, the method is highly sensitive to variations in viewpoint; discrepancies in the computation of local bolt normal vectors diminish histogram similarity and lead to a significant decrease in registration accuracy. Moreover, this approach lacks adequate real-time performance.

The Iterative Closest Point (ICP) method exhibits limited adaptability. In tunnel environments, the presence of mud obstructions and deviations in viewpoint diminish the overlap ratio of point clouds, causing ICP to be susceptible to local minima or divergence. This often leads to the failure to produce valid coordinate outputs.

The Predator method, while optimized for scenarios with minimal overlap, necessitates extensive training on thousands of bolt samples across diverse occlusion and viewing conditions. However, in actual tunnel environments, the levels of occlusion and the patterns of mud adhesion on bolts can differ greatly, complicating the ability to encompass all potential cases and increasing the likelihood of overfitting. Furthermore, the method’s inference time is prolonged, which does not satisfy the real-time requirements of the original operating system.

In contrast, the method introduced in this study exhibits enhanced adaptability. Throughout 12 tunnel test scenarios, the mean absolute localization error was consistently low, with no significant anomalies observed. Additionally, the approach achieved rapid inference speeds, meeting real-time operational demands while necessitating minimal preprocessing efforts. The findings validate that the SIFT feature-based visual localization technique offers high precision and robust stability for bolt indirect localization method within the integrated disc cutter system.

### 3.5. Performance Evaluation of Real-Time Capability

To quantitatively demonstrate the feasibility of the proposed method in practical engineering scenarios, we systematically recorded the total runtime of the entire program execution process and the real-time performance metrics of each core link, the details are shown in Table 9 below. These statistical metrics serve three key purposes: (1) directly verifying whether the method meets the real-time requirements for the localization of shield machine cutter-changing robots; (2) quantifying the time consumption of each technical link, such as segmentation, coordinate transformation, SIFT feature extraction, and RANSAC-based pose estimation, to identify performance bottlenecks and provide a data foundation for subsequent algorithm optimization; (3) clarifying the hardware resource occupation of the method during operation, thereby proving its adaptability to the embedded computing environment of shield machine cutter-changing robots—characterized by low CPU load and controllable GPU resource demand.

The statistical results show that the total runtime of the proposed method is 9.665 s, with the RANSAC-based pose estimation and SIFT feature projection being the main time-consuming links, accounting for 77.1% of the total time. The average CPU load is 8.54%, and the GPU load is low for single-frame processing, indicating that the method has low hardware resource requirements and can stably run in the embedded environment of the cutter changing robot.

## 4. Conclusions

This research introduces a bolt indirect localization technique that utilizes image-point cloud fusion to tackle localization difficulties encountered during tunnel disc cutter replacement operations. These challenges arise from confined spaces, poor lighting, and mud-water contamination, which result in incomplete point clouds and target occlusion. A SCMamba-YOLO model, which incorporates OS2 Blocks for multi-level feature extraction, is developed to achieve precise segmentation of feature planes. Additionally, a non-overlapping point cloud registration method based on image-point cloud fusion is established. Through extrinsic calibration, a coordinate mapping between the depth and texture cameras is created, enabling the application of segmented masks for point cloud filtering and corner extraction. The SIFT-based feature matching method, combined with RANSAC optimization, is employed to compute the essential matrix and finalize pose calibration, followed by coordinate transformation to determine bolt coordinatess. This approach addresses the shortcomings of traditional ICP algorithms, such as their vulnerability to local minima, and reduces the reliance of deep learning methods on extensive datasets, thereby improving the robustness of non-overlapping point cloud registration. Experimental findings indicate that, under optimal conditions, the average localization error is less than 1.98 mm, with a mean Z-axis deviation of only 0.32 mm. In simulated tunnel environments, the SIFT-based localization technique consistently achieves an average error below 2 mm across varying illumination, viewing angles, and distances, confirming its adaptability and robustness in complex operational settings.

## Figures and Tables

**Table 1:** Comparison of Point Cloud Registration Methods.

Method Category	Representative Methods	Limitations and Characteristics
Traditional Point Cloud Registration	ICP, FPFH	Relies on point cloud geometric features, low overlap or mud contamination leads to frequent registration failures
Deep Learning-based Point Cloud Registration	PointDiformer, BiEquiformer	Requires large-scale samples, data-dependent, slow inference, prone to overfitting in tunnel scenarios. In the case of non-overlapping point clouds, point cloud registration methods fail.
indirect image-point cloud fusion straegy(our method)	–	Integrated segmentation-registration, strong anti-interference, low positioning error, supports real-time operation

**Table 2:** Distribution of images by environmental factors in HCB-Dataset.

Group	Well-Light	Low-Light	No Contamination	Mild Slurry Contamination	Moderate Slurry Contamination	Image Count
1	√	×	√	×	×	50
2	×	√	√	×	×	50
3	√	×	×	√	×	30
4	×	√	×	√	×	30
5	√	×	×	×	√	20
6	×	√	×	×	√	20

**Table 3:** Results of segmentation on the HCBDataset instance segmentation benchmark.

Methods	mAP50 (%)	mAP50-95 (%)	Params	FLOPs	Latency
Mamba-YOLO-T	**86.4**	**77.1**	5.8	13.2 G	1.5 ms
Yolov11-n-segment [28]	**86.2**	**76.8**	2.9 M	10.4 G	1.84 ms
RTMDet-Ins-T [29]	–	**78.7**	5.6 M	23.6 G	1.89 ms
RTMDet-Ins-S [29]	–	**80.8**	10.2 M	43.0 G	2.26 ms
YOLOv11-s-segment [28]	**88.2**	**79.6**	10.1 M	35.5 G	2.95 ms
SparseInst-R50 [30]	**87.1**	**77.3**	31.6 M	99.1 G	11.60 ms
SCMamba-YOLO	**90.7**	**82.2**	5.92 M	13.36 G	1.51 ms

**Table 4:** Ablation Test Results.

Group	ASPP	OS2 Block (Without SCSA)	OS2 Block (with SCSA)	mAP50 (%)	mAP50-95 (%)
1				0.864	0.771
2	√	×	×	0.871	0.793
3	×	√	×	0.883	0.802
4	×	×	√	0.894	0.809
5	√	√	×	0.887	0.804
6	√	×	√	0.907	0.822

**Table 5:** Experimental data parameters.

ID	Position	Calculated Bolt Coordinates (mm)	Actual Bolt Coordinates (mm)	Error (mm)
a	Upper	[−170.8, 168.6, 472.0]	[−171.9, 167.6, 472.0]	[0.9, 1.00, 0.0]
	Lower	[−170.4, −163.2, 449.2]	[−171.1, −164.1, 449.4]	[0.7, 0.9, −0.2]
b	Upper	[−173.6, 168.9, 465.4]	[−175.6, 168.8, 465.8]	[2.0, 0.1, −0.4]
	Lower	[−173.3, −164.6, 441.6]	[−174.8, −162.9, 442.9]	[1.5, −1.7, −1.3]
c	Upper	[−174.6, 181.4, 516.0]	[−174.6, 180.7, 515.1]	[0.0, 0.7, 0.9]
	Lower	[−174.6, −151.7, 499.6]	[−174.8, −151.4, 499.6]	[0.2, −0.3, 0.00]
d	Upper	[−166.8, 182.9, 529.4]	[−167.3, 181.5, 528.5]	[0.5, 1.4, 0.9]
	Lower	[−166.7, −151.2, 514.8]	[−167.0, −150.7, 514.4]	[0.3, −0.5, 0.4]
e	Upper	[−177.1, 182.9, 532.0]	[−178.1, 182.5, 532.2]	[1.0, 0.4, −0.2]
	Lower	[−176.8, −151.2, 516.1]	[−178.1, −149.6, 517.3]	[1.3, −1.6, −1.2]
f	Upper	[−188.3, 182.4, 529.8]	[−188.6, 181.6, 529.1]	[0.3, 0.8, 0.7]
	Lower	[−187.3, −151.5, 513.9]	[−188.0, −150.6, 514.4]	[0.7, −0.9, −0.5]

**Table 6:** Experimental data parameter table.

ID	Position	Calculated Bolt Coordinates (mm)	True Bolt Coordinates (mm)	Error (mm)
a	Upper	[−80.1, 180.7, 660.8]	[−80.3, 179.4, 658.9]	[0.2, 1.3, 1.9]
	Lower	[−74.6, −151.4, 659.2]	[−73.0, −151.4, 658.4]	[−1.6, 0.0, 0.8]
b	Upper	[−210.4, 182.2, 619.5]	[−209.5, 181.7, 619.3]	[−0.9, 0.5, 0.2]
	Lower	[−204.1, −149.9, 619.0]	[−204.8, −149.3, 620.2]	[0.7, −0.6, −1.2]
c	Upper	[−197.4, 152.2, 741.6]	[−198.8, 151.5, 740.4]	[1.4, 0.7, 1.2]
	Lower	[−172.9, −178.6, 725.0]	[−172.7, −179.8, 725.2]	[−0.2, 1.2, −0.2]
d	Upper	[−182.1, 168.5, 604.4]	[−181.2, 170.5, 605.3]	[−0.9, −2.0, −0.9]
	Lower	[−171.6, −163.4, 600.2]	[−172.9, −163.2, 598.5]	[1.3, −0.2, 1.7]
e	Upper	[−182.9, 169.1, 605.4]	[−181.2, 170.5, 605.3]	[−1.7, −1.4, 0.1]
	Lower	[−172.3, −162.9, 600.1]	[−172.9, −163.2, 598.5]	[0.6, 0.3, 1.6]
f	Upper	[−182.4, 169.9, 606.1]	[−181.2, 170.5, 605.3]	[−1.2, −0.6, 0.8]
	Lower	[−172.8, −162.1, 600.0]	[−172.9, −163.2, 598.5]	[0.1, 1.1, 1.5]
g	Upper	[−182.9, 170.2, 605.3]	[−181.2, 171.2, 604.8]	[−1.7, −1.0, 0.5]
	Lower	[−172.2, −161.7, 600.2]	[−171.0, −163.1, 598.4]	[−1.2, 1.4, 1.8]
h	Upper	[−169.5, 177.8, 622.4]	[−168.1, 176.2, 622.4]	[−1.4, 1.6, 0.0]
	Lower	[−162.2, −154.2, 622.9]	[−162.0, −155.8, 622.3]	[−0.2, 1.6, 0.6]
i	Upper	[−182.3, 170.1, 605.8]	[−181.2, 171.2, 604.8]	[−1.1, −1.1, 1.0]
	Lower	[−172.6, −161.8, 599.9]	[−171.0, −163.1, 598.4]	[−1.6, 1.3, 1.5]
j	Upper	[−168.2, 175.4, 420.1]	[−167.8, 173.4, 418.4]	[−0.4, 2.0, 1.7]
	Lower	[−158.0,−156.6, 416.4]	[−158.8, −154.8, 415.4]	[0.8, −1.8, 1.0]
k	Upper	[−181.8, 169.9, 606.1]	[−181.2, 170.5, 605.3]	[−0.6, −0.6, 0.8]
	Lower	[−172.8, −162.1, 600.1]	[−172.9, −163.2, 598.5]	[0.1, 1.1, 1.6]
l	Upper	[−193.3, 191.3, 921.7]	[−193.9, 192.2, 923.6]	[0.6, −0.9, −1.9]
	Lower	[−197.2, −140.8, 918.0]	[−196.4, −139.5, 917.8]	[−0.8, −1.3, 0.2]

**Table 7:** Experimental data parameter table.

**ID**	**Position**	**AKAZE-Registered** **Bolt Coordinates**	**Bolt Error**	**ORB-Registered Bolt** **Coordinates**	**Bolt Error**
a	Upper	[−80.6, 176.5, 659.6]	[−0.3, −2.9, 0.7]	[−78.3, 178.1, 659.5]	[2.0, −1.3, 0.6]
	Lower	[−73.3, −154.3, 657.7]	[−0.3, −2.9, −0.7]	[−71.5, −152.7, 658.3]	[1.5, −1.3, −0.1]
b	Upper	[−211.3, 181.5, 618.7]	[−1.8, −0.2, −0.6]	[−21.0, 183.1, 618.4]	[−1.5, 1.4, −0.9]
	Lower	[−206.2, −149.5, 619.7]	[−1.4, −0.2, −0.5]	[−206.4, −147.8, 620.0]	[−1.6, 1.5, −0.2]
c	Upper	[−197.8, 105.2, 748.7]	[1.0, −46.3, 8.3]	[−202.9, 188.4, 730.7]	[−4.1, 36.9, −9.7]
	Lower	[−168.6, −224.4, 713.6]	[4.1, −44.6, −11.6]	[−187.9, −144.0, 729.4]	[−15.2, 35.8, 4.2]
d	Upper	[−186.9, 172.5, 603.0]	[−5.7, 2.0, −2.3]	[−545.3, 339.1, 123.0]	[−364.1, 168.6, −482.3]
	Lower	[−178.3, −161.3, 597.5]	[−5.4, 1.9, −1.0]	[−611.8, 20.6, 198.0]	[−438.9, 183.8, −400.5]
e	Upper	[−182.5, 170.8, 604.8]	[−1.3, 0.3, −0.5]	[−92.7, 177.9, 622.6]	[88.5, 7.4, 17.3]
	Lower	[−174.5, −162.9, 598.2]	[−1.6, 0.3, −0.3]	[−74.0, −155.4, 620.3]	[98.9, 7.8, 21.8]
f	Upper	[−181.7, 171.2, 605.0]	[−0.5, 0.7, −0.3]	[−181.6, 170.5, 605.2]	[−0.4, 0.0, −0.1]
	Lower	[−173.5, −162.6, 598.5]	[−0.6, 0.6, 0.0]	[−172.3, −163.3, 598.7]	[0.6, −0.1, 0.2]
g	Upper	[−182.4, 169.9, 604.8]	[−1.2, −1.3, 0.0]	[−180.4, 184.4, 601.1]	[0.8, 13.2, −3.7]
	Lower	[−173.0, −164.4, 597.4]	[−2.0, −1.3, −1.0]	[−177.6, −150.1, 599.9]	[−6.6, 13.0, 1.5]
h	Upper	[−167.3, 181.5, 621.1]	[0.8, 5.3, −1.3]	[−206.3, 126.0, 623.0]	[−38.2, −50.2, 0.6]
	Lower	[−162.6, −150.5, 623.4]	[−0.6, 5.3, 1.1]	[−159.8, −202.5, 609.0]	[2.2, −46.7, −13.3]
i	Upper	[−180.9, 174.0, 604.1]	[0.3, 2.8, −0.7]	[−177.1, 185.2, 601.8]	[4.1, 14.0, −3.0]
	Lower	[−171.1, −160.4, 599.1]	[−0.1, 2.7, 0.7]	[−173.9, −149.3, 601.2]	[−2.9, 13.8, 2.8]
j	Upper	[−167.2, 174.4, 418.3]	[0.6, 1.0, −0.1]	[−179.6, 163.5, 417.6]	[−11.8, −9.9, −0.8]
	Lower	[−158.5, −153.8, 415.9]	[0.3, 1.0, 0.5]	[−173.4, −164.6, 405.6]	[−14.6, −9.8, −9.8]
k	Upper	[−181.7, 171.2, 605.0]	[−0.5, 0.7, −0.3]	[−181.6, 170.5, 605.2]	[−0.4, 0.0, −0.1]
	Lower	[−173.5, −162.6, 598.5]	[−0.6, 0.6, 0.0]	[−172.3, −163.3, 598.7]	[0.6, −0.1, 0.2]
l	Upper	[−152.5, 97.6, 945.9]	[41.4, −94.6, 22.3]	[−163.4, 177.4, 932.4]	[30.5, −14.8, 8.8]
	Lower	[−152.5, 97.6, 945.9]	[43.9, 237.1, 28.1]	[−163.4, 177.4, 932.4]	[33.0, 316.9, 14.6]

**Table 9:** Real-time performance metrics for program runtime.

Link	Corresponding Data
Segmentation	2.118 s
Coordinate transformation	0.097 s
SIFT + Projection	3.199 s
RANSAC	4.251 s
Total runtime	9.665 s
Average CPU load	8.54%

**Table 8:** Matching results of three feature matching algorithms under conditions a to l.

	a	b	c	d	e	f	j	h	i	g	k	l
AKAZE	64	95	94	111	164	191	228	179	101	104	142	140
ORB	363	452	542	427	698	849	1074	861	563	410	701	588
SIFT	475	591	678	630	991	953	1119	968	669	869	1045	927

## Data Availability

The data that supports the findings of this study are available upon reasonable request from the authors.
